# Therapeutic response to exclusive enteral nutrition correlates with suppressed IL-17 signaling activity and diminished IL-1β secretion in pediatric Crohn's disease

**DOI:** 10.3389/fnut.2026.1901066

**Published:** 2026-09-14

**Authors:** Wenjuan Tang, Yuhuan Wang, Zifei Tang, Cuifang Zheng, Jieru Shi, Yingying Meng, Ying Huang

**Affiliations:** Department of Gastroenterology, Pediatric Inflammatory Bowel Disease Research Center, National Children's Medical Center, Children's Hospital of Fudan University, Shanghai, China

**Keywords:** Crohn's disease, EEN, IL-17, IL-1β, pediatric

## Abstract

**Background:**

To identify molecular correlates associated with therapeutic outcomes of exclusive enteral nutrition (EEN) in pediatric Crohn's disease (PCD).

**Methods:**

This prospective observational cohort study recruited 27 treatment-naive pediatric patients with endoscopically active PCD. Intestinal mucosal tissues were collected via endoscopy at baseline and after EEN treatment. RNA sequencing, principal component analysis (PCA), protein–protein interaction (PPI) analysis, and quantitative real-time polymerase chain reaction (qRT-PCR) were performed to identify and validate differentially expressed genes (DEGs). Patients were classified into mucosal healing [MH, Simple Endoscopic Score for Crohn's Disease (SES-CD) < 3, *n* = 13] and non-mucosal healing (NMH, SES-CD ≥ 3 with ulcers, *n* = 14) groups according to post-treatment SES-CD.

**Results:**

PCA revealed partial transcriptomic segregation between the MH and NMH groups after EEN. Bioinformatic enrichment analysis identified the IL-17 signaling pathway as the most significantly enriched pathway, encompassing nine core DEGs. Integrated qRT-PCR validation and PPI network analysis pinpointed IL-1β as the key target gene. Correlation analysis demonstrated that intestinal mucosal IL-1β expression was moderately and positively correlated with SES-CD scores (*P* = 0.001, *r* = 0.42, 95% CI: 0.17–0.63), Lewis scores (*P* < 0.001, *r* = 0.65, 95% CI: 0.41–0.79), and weighted Pediatric Crohn's Disease Activity Index (wPCDAI) values (*P* < 0.001, *r* = 0.51, 95% CI: 0.26–0.69). Moreover, compared with the NMH group, the MH group exhibited lower mucosal expression levels of IL-17A [MH: 0.50 (1.42) vs. NMH: 2.58 (2.05), *P* = 0.03], IL-17RA (MH: 0.95 ± 0.37 vs. NMH: 1.34 ± 0.53, *P* = 0.04), and IL-17RB [MH: 1.18 (0.54) vs. NMH: 1.64 (1.32), *P* < 0.01] after EEN treatment.

**Conclusions:**

EEN attenuates intestinal inflammation in PCD, and this therapeutic response may associate with suppressed IL-17 signaling activity and reduced IL-1β secretion.

## Introduction

Crohn's disease (CD), whose precise pathogenesis remains elusive, has emerged as a growing global health concern among children and adolescents (1). Pediatric CD (PCD) is characterized by more severe clinical manifestations, higher vulnerability to surgery-requiring complications, and even life-threatening outcomes, with earlier onset correlating with greater disease severity (1). Clinically, mucosal healing (MH) serves as the primary therapeutic target for PCD. However, achieving and sustaining MH remains extremely challenging due to the disease's complexity and high recurrence rate. Our center has previously reported that the MH rate reached 30.6% after infliximab-induced remission (2), and 46% after exclusive enteral nutrition (EEN)-induced remission, which declined to 34% at the 1-year follow-up (3). Furthermore, marked inter-individual heterogeneity in therapeutic responses is commonly observed even in patients receiving identical treatment regimens. For instance, patients may exhibit either responsiveness or non-responsiveness to infliximab treatment (4). Similarly, EEN is associated with issues such as significant variability in treatment outcomes (5). EEN, a therapeutic regimen comprising exclusive formula feeding for 6–8 weeks, is recommended as first-line induction therapy for remission in PCD (6). However, the specific molecular factors linked to EEN efficacy remain unclear, and relevant research is still scarce (7, 8). Existing studies mainly focus on overall alterations of the gut microbiota or the regulation of host immune responses, exemplified by widespread changes in the Th17 cell population (5, 7–9), lacking targeted transcriptomic evidence from human intestinal tissue to distinguish molecular profiles between responders and non-responders. This critical knowledge gap limits our capacity to predict EEN efficacy and design personalized nutritional interventions for PCD.

Th17 cells are generally regarded as the primary source of IL-17 and abnormal activation of the IL-17 pathway is closely associated with intestinal inflammation in CD (10–12). Notably, Th17 cells persist at higher frequencies even in MH tissue when compared with non-CD controls, and this reflects persistently disturbed microbiome-host interaction (12). Th17 cells and gut microbiota share a close bidirectional regulatory relationship: gut microbiota governs Th17 cell differentiation, and Th17 cells reciprocally reshape gut microbiota (13). Modulation of the gut microbiota has been hypothesized as one key factor linked to EEN therapeutic effects for PCD. A previous study has shown that EEN could dampen the Th17 immune response by eliminating mechanical stress in mice (9), nevertheless, human mucosal transcriptomic data validating this connection during clinical EEN treatment are still absent. Accordingly, we hypothesized that the IL-17 signaling pathway may be closely associated with the therapeutic response of EEN in children with PCD. In this prospective observational cohort study, we divided PCD patients into an MH group and a non-mucosal healing (NMH) group based on whether MH was achieved endoscopically at EEN completion. Through RNA-seq detection and subsequent qRT-PCR validation in intestinal mucosa, we identified that the reduced IL-1β secretion and altered IL-17 signaling pathway activity are correlated with the therapeutic response of EEN in PCD.

## Method

### Study design, participants and sample size calculation

This prospective observational cohort study was conducted at the Children's Hospital of Fudan University between September 2021 and December 2024. The inclusion criteria were as follows: (1) aged 6–18 years; (2) newly diagnosed and treatment-naive PCD confirmed by comprehensive clinical manifestations, endoscopic findings, histological examinations and imaging evaluations according to the Porto criteria (14); (3) endoscopically active disease defined as the Simple Endoscopic Score for CD (SES-CD) ≥ 3 accompanied by intestinal ulcers; (4) EEN administered as the sole induction therapy. Exclusion criteria included: congenital genetic syndromes, acute intestinal infections, concomitant use of corticosteroids, immunosuppressive or biological agents, incomplete endoscopic and sample data, and poor EEN compliance.

The sample size was determined a priori via power analysis using G^*^Power 3.1 software (Heinrich-Heine-Universität Düsseldorf, Düsseldorf, Germany), based on preliminary experimental data of mucosal IL-1β expression (fold change = 1.8, SD = 0.6). We set the significance level α = 0.05 and statistical power of 0.8 (β = 0.2). The calculation indicated that a minimum of 22 participants were required. To account for potential loss-to-follow-up or sample-quality-related drop-outs, we aimed to recruit 27 patients; a total of 27 patients were finally enrolled in the present study.

### EEN treatment protocol

All patients received EEN for at least 6 weeks with polymeric or oligomeric enteral formula, which provided more than 110–120% of the individual estimated daily energy requirements based on age and sex. The actual EEN treatment duration ranged from 6 to 19.3 weeks in our cohort. No additional oral food or beverages were permitted throughout the entire treatment period; only a small amount of water ( ≤ 100 mL/d) was allowed. EEN compliance was assessed by pediatric gastroenterologists according to dietary logs, formula consumption records, and clinical follow-up interviews; poor compliance was defined as regular intake of forbidden oral food/beverages or formula intake below 80% of prescribed daily energy requirement, which served as an exclusion criterion.

### Endoscopic evaluation and grouping

All patients underwent standard colonoscopy at baseline (pre-EEN initiation) and post-EEN treatment. Endoscopic images were independently assessed by two experienced pediatric gastroenterologists who were blinded to all clinical and treatment information, and the SES-CD was systematically calculated for each participant. Inter-rater agreement for SES-CD scoring between the two endoscopists was evaluated, with a Cohen's kappa coefficient of 0.81 indicating substantial agreement. Based on post-EEN SES-CD values, patients were stratified into two groups: the MH group (SES-CD < 3) (6) and the NMH group (SES-CD ≥ 3, with persistent ulceration).

### Data and sample collection

Participants' medical records were extracted from the hospital database, covering the period from baseline (within 1 week before EEN initiation) to post-EEN treatment (within 1 week after EEN completion). The collected data included patient characteristics, disease activity markers [Paris Classification, Lewis score, weighted Pediatric Crohn's Disease Activity Index (wPCDAI), fecal calprotectin (FCP), and erythrocyte sedimentation rate (ESR)], and nutritional indicators [weight, height, serum albumin (ALB) level, and hemoglobin (Hgb) level]. Approximately 5–8 mg of intestinal mucosal biopsy tissue (combining two individual biopsy fragments from the target site) was obtained per subject-time-point. Intestinal mucosal tissues were collected endoscopically at two time points: baseline (MH-1/NMH-1) and post-EEN (MH-2/NMH-2). Immediately after collection, all tissue samples were stored in RNALater (Beyotime, Cat. No. R0118), snap-frozen in liquid nitrogen, and preserved at −80 °C until subsequent analysis. At baseline, mucosal tissues were sampled from areas adjacent to intestinal ulcers. After EEN, for patients who achieved MH, tissues were collected from the same intestinal segment as that at baseline, for those patients without MH, tissues were sampled from regions adjacent to persistent ulcers, with priority given to the original baseline segment when feasible.

### RNA extraction

Total RNA was extracted from the tissue using TRIzol reagent (Thermo Scientific, Waltham, MA, USA) according to the manufacturer's instructions. On-column DNase I digestion was performed during RNA purification to eliminate genomic DNA contamination. RNA quality was then determined by an Agilent 5300 Bioanalyzer (Agilent Technologies, Santa Clara, CA, USA) and quantified using the ND-2000 spectrophotometer (NanoDrop Technologies, Wilmington, DE, USA). The acceptable total RNA concentration threshold was set at ≥100 ng/μL. Only high-quality RNA samples (OD_260/280_ = 1.8–2.2, OD_260/230_ ≥ 2.0, RQN ≥ 6.5, 28S:18S ≥ 1.0, >1 μg) were used for further analysis. These quality cut-offs were predefined to ensure sufficient RNA integrity, minimal protein-polysaccharide contamination, and adequate input mass for stranded mRNA library construction for RNA-seq; samples failing any of these criteria were excluded from subsequent sequencing and qRT-PCR analyses.

### Transcriptomic profiling and bioinformatic analysis

#### Transcriptomic analysis

RNA purification, reverse transcription, library construction and sequencing were performed according to the manufacturer's instructions (Illumina, San Diego, CA). The RNA-seq transcriptome library was prepared following the Illumina^®^ Stranded mRNA Prep Ligation protocol (Illumina, San Diego, CA, USA) using 1ug high-quality total RNA. Briefly, messenger RNA (mRNA) was isolated according to the polyA selection method by oligo (dT) beads and then fragmented with fragmentation buffer. Subsequently, double-stranded cDNA was synthesized using a SuperScript double-stranded cDNA synthesis kit (Invitrogen, Waltham, MA, USA) with random hexamer primers (Illumina, San Diego, CA, USA). The synthesized cDNA was then subjected to end-repair, phosphorylation and “A” base addition according to Illumina's library construction protocol. Libraries were size-selected for cDNA target fragments of 300 bp on 2% Low Range Ultra Agarose, followed by PCR amplification using Phusion DNA polymerase (New England Biolabs, Ipswich, MA, USA) for 15 PCR cycles. After quantification by Qubit 4.0, fluorometer (Thermo Scientific, Waltham, MA, USA), the paired-end RNA-seq sequencing library was sequenced with the NovaSeq X Plus sequencer (Illumina, San Diego, CA, USA, 2 × 150 bp read length).

#### Raw sequencing data quality control and read mapping

Raw paired-end fastq reads were preprocessed and quality-filtered using fastp software (v0.23.2) with standardized quality-control thresholds: sequencing adapters were trimmed; 3′-terminal bases with Phred quality < 20 were clipped; reads containing over 10% ambiguous N bases or remaining length < 20 bp after trimming were discarded. Sequencing quality criteria required Q20 ≥ 90% and Q30 ≥ 85% for each sample to proceed to downstream analysis. Comprehensive read-quality metrics were calculated for all filtered clean reads, with the average Q20 value reaching 97.6% and average Q30 value reaching 93.2% across all samples. High-quality clean reads were aligned to the human reference genome assembly GRCh38.p13 using GENCODE v44 genome annotation for gene-model reference, in strand-specific orientation via HISAT2 (v2.1.0) and the overall average genome mapping rate of all samples was 94.8%. Transcript assembly was implemented with StringTie (v2.2.1) using a reference-guided mode. Each sequencing library generated an average of 42 million paired-end raw reads, corresponding to an average transcriptome sequencing depth of 12 × .All downstream transcriptome quantification and bioinformatic analyses were executed on the Majorbio Cloud Platform (Majorbio Bio-Pharm Technology Co., Ltd., Shanghai, China).

#### Transcript quantification and principal component analysis (PCA)

Gene transcript abundance was normalized and quantified with RSEM (v1.3.3) using the TPM-based normalization procedures (transcripts per million) to eliminate technical bias derived from variable sequencing depth and gene length across different samples. For DESeq2 differential analysis, raw read counts (not TPM values) were used as input, with size-factor normalization applied internally within DESeq2 to correct for library-size differences. To globally assess transcriptional heterogeneity between patient subgroups at baseline and post-EEN timepoints, PCA was performed on log_2_-transformed TPM expression matrices of all detected protein-coding transcripts. No additional filtering was applied prior to PCA computation; sample expression profiles were directly input for dimensionality reduction to visualize overall transcriptome separation between MH and NMH cohorts. To statistically validate the transcriptomic separation shown in PCA plots, permutational multivariate analysis of variance (PERMANOVA) was used to quantify transcriptional variation and test for significant differences between MH and NMH groups at baseline and post-EEN. This analysis was performed using the adonis2 function of R package vegan_2.7-5 with 999 permutations, with grouping (MH vs. NMH) specified as the main explanatory variable in the model.

#### Differential expressed genes (DEGs) screening

To identify DEGs between the two sample groups, the expression level of each transcript was calculated using the TPM method. RSEM (v1.3.3) software was employed to quantify gene abundances, and differential expression analysis was performed using DESeq2 R package (v1.36.0). The DESeq2 design formula ~ group was applied to model the effect of patient group (MH-2 vs NMH-2; MH-1 vs. MH-2). Genes with |log2FC| ≥ 1 and FDR ≤ 0.05 (determined by DESeq2) were considered significantly differentially expressed. The Benjamini-Hochberg (BH) procedure was implemented within DESeq2 for multiple-testing correction to control false-discovery rate.

#### Kyoto encyclopedia of genes and genomes (KEGG) pathway enrichment analysis

KEGG pathway enrichment of overlapping DEGs was conducted using KOBAS (v3.0). Fisher's exact test with BH multiple testing correction was adopted, and pathways with corrected *P*-value ≤ 0.05 were regarded as significantly enriched functional pathways.

#### Protein-protein interactions (PPIs) network construction

The Search Tool for the Retrieval of Interacting Genes/Proteins (STRING database, version 11.5, EMBL-EBI, Cambridge, UK) was utilized to construct the interaction network of overlapping DEGs. Only protein interaction pairs with a confidence score > 0.4 were retained for network visualization and topological analysis via Cytoscape software (v3.10.4, Institute for Systems Biology, Seattle, WA, USA). Node degree centrality was calculated to screen core hub genes within the PPI network.

### Quantitative reverse transcription polymerase chain reaction (qRT-PCR*)*

qRT-PCR was performed using the Universal Blue SYBR Green qRT-PCR master mix (Servicebio, Wuhan, Hubei, China). The PCR conditions were set as follows: 95 °C for 30 s, followed by 40 cycles of 95 °C for 5 s and 60°C for 30 s, then a melting curve analysis from 65°C to 95°C. GAPDH was used as the reference gene for all target genes. Relative gene expression levels were analyzed by the 2^(^−ΔΔCt) method. Each patient-derived mucosal sample represented one biological replicate; every qRT-PCR assay was run in three independent technical replicates to reduce pipetting and reaction variation. The primers used in this study are listed in Supplementary Table 1, which summarizes the full forward and reverse oligonucleotide sequences for all target genes and the reference gene GAPDH applied in qRT-PCR amplification.

### Statistical analysis

All statistical analyses were performed using GraphPad Prism Software version 10.0 (GraphPad Software, San Diego, CA, USA). The D'Agostino–Pearson omnibus test was applied to evaluate the normality of continuous variables. Normally distributed data were presented as mean ± standard deviation, with between-group differences compared via Student's *t*-test. Non-normally distributed data were described as median and interquartile range (IQR, 25th−75th percentile), and group comparisons were performed using the Mann–Whitney *U* test. Categorical variables were summarized as numbers and percentages, and intergroup discrepancies were assessed by Fisher's exact test. Spearman's rank correlation analysis was used to explore correlation relationships. No extra multiple-test adjustment was implemented for these pre-specified clinical comparisons, since all tests were hypothesis-driven and restricted to our core predefined endpoints; universal multiple-test correction would substantially increase the risk of false-negative findings in this small-sized pediatric cohort. A two-sided *P* value ≤ 0.05 was defined as statistically significant.

## Results

### Characteristics of enrolled patients

In total, 65 patients were assessed for eligibility and initiated exclusive enteral nutrition monotherapy. Of these assessed candidates, 38 patients were excluded for predefined individual reasons (detailed categorized exclusion causes with patient numbers are presented in Figure 1), leaving 27 treatment-naïve patients with endoscopically active PCD included for final analysis. Of the 27 included patients, 13 patients were assigned to the MH group and 14 patients to the NMH group after EEN treatment (Figure 1). Baseline demographic data, disease activity indices, nutritional status and endoscopic scores were comparable between the two groups, with no statistically significant differences observed (all *P* > 0.05, Table 1).

**Figure 1:**
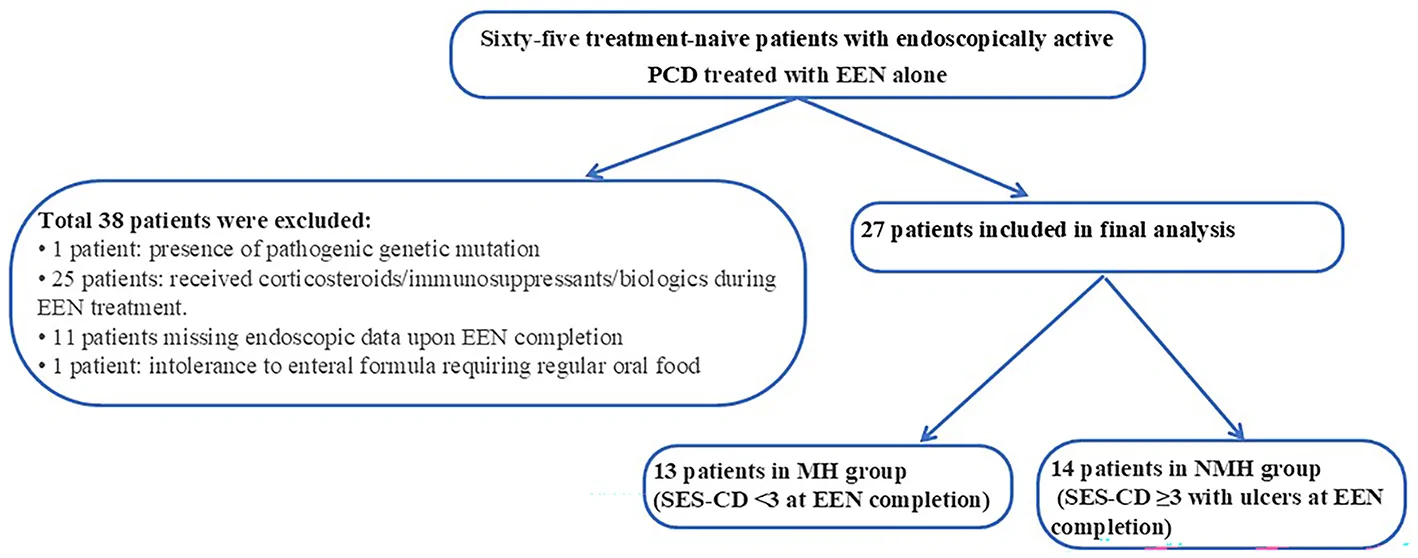
Flow diagram of patient enrollment in this study. PCD, pediatric Crohn's disease; EEN, exclusive enteral nutrition; MH, mucosal healing; NMH, non-mucosal healing; SES-CD, simple endoscopic score for CD.

**Table 1:** Characteristics and clinical information for patients enrolled in this study.

Variables	MH (*n* = 13)			NMH (*n* = 14)				
	Baseline	Ending	*P* ^a^	Baseline	Ending	*P* ^a^	*P* ^b^	*P* ^c^
AOD (y)	12.26 ± 1.70^1^		10.88 ± 1.95		0.06			
AOO (y)	11.84 ± 1.87		10.64 ± 2.01		0.12			
Male *n* (%)	8 (61.54%)		10 (71.43%)		0.69			
DD (M)	2.00 (3.00)^2^		2.50 (5.25)		0.86			
ED (W)	10.60 (3.70)		9.65 (1.60)		0.56			
DEI (kcal/d)	2,400.00 ± 495.00		2,104.00 ± 264.90		0.06			
SES-CD	11.92 ± 6.42	0.00 (2.00)	< 0.01	10.21 ± 6.28	4.00 (3.25)	0.03	0.49	< 0.01
Lewis score	661.00 (1,588.00)	0.0 (233.00)	< 0.01	506.00 (781.20)	116.50 (399.30)	< 0.01	0.34	0.42
wPCDAI	51.25 (35.00)	7.50 (15.00)	< 0.01	42.86 ± 11.80	7.50 (12.50)	< 0.01	0.87	0.56
FCP(μg/g)	486.60 ± 310.30	40.74 (115.41)	< 0.01	294.90 ± 185.80	42.11 (292.45)	0.01	0.06	0.98
ESR (mm/h)	65.92 ± 34.17	19.00 (13.50)	< 0.01	77.69 ± 27.56	24.00 (24.25)	< 0.01	0.34	0.37
Hgb (g/L)	117.90 ± 19.38	134.40 ± 14.28	0.02	119.80 ± 18.09	131.50 (9.50)	0.04	0.80	0.36
ALB (g/L)	38.06 ± 5.57	43.77 ± 2.28	< 0.01	36.53 ± 6.50	44.03 ± 3.35	< 0.01	0.55	0.82
WG^3^ (kg)	2.73 ± 4.57			3.21 ± 2.68				0.73
**Disease location at baseline**								
L1 *n* (%)	2 (15.38%)			4 (28.75%)			0.64	
L1+L4 *n* (%)	2 (15.38%)			3 (21.43%)			>0.99	
L2 *n* (%)	3 (23.08%)			2 (14.29%)			0.64	
L2+L4 *n* (%)	2 (15.38%)			1 (7.14%)			0.60	
L3 *n* (%)	8 (61.54%)			8 (57.14%)			>0.99	
L3+L4 *n* (%)	7 (53.85%)			7 (50.00%)			>0.99	
L4 n (%)	11 (84.62%)			12 (85.71%)			>0.99	
B1/B2 *n*	13/0			13/1			>0.99	
P1 *n* (%)	5 (38.46%)			1 (7.14%)			0.08	

Baseline: within 1 week before EEN initiation; Ending: within 1 week after EEN completion. MH, mucosal healing; NMH, non-mucosal healing; AOD, age of diagnosis; AOO, age of onset; DD, Disease duration; ED,EEN duration; EEN, exclusive enteral nutrition; DEI, Daily energy intake; SES-CD, simple endoscopic score for Crohn's disease; wPCDAI, weighted pediatric Crohn's disease activity index; FCP, fecal calprotectin; ESR, erythrocyte sedimentation rate; Hgb, hemoglobin; ALB, serum albumin level; WG: weight gain; y, year; M, month; W, week. *P*^*a*^: Baseline vs Ending. *P*^*b*^: MH vs. NMH at baseline. *P*^*c*^: MH vs. NMH at EEN ending. 1, The normally distributed quantitative variables were expressed as means±SD, and Student t-tests were used to analyze the differences. 2, The skewed quantitative variables were expressed as medians (interquartile range), and Mann-Whitney test was used to analyze the differences. 3, WG value = Ending–Baseline.

### Whole transcriptomic profiling of study subjects

RNA-sequencing was performed to conduct whole-transcriptome analysis in patients from both groups. Sequencing data preprocessing identified a total of 274,966 transcripts. Baseline principal component analysis showed no evident transcriptional distinction between MH and NMH samples (Figure 2A). By contrast, a partial separation trend emerged upon completion of EEN therapy, alongside an increased explanatory proportion of the first principal component (Figure 2B), suggesting transcriptomic alterations correlated with clinical disease amelioration following EEN treatment.

**Figure 2:**
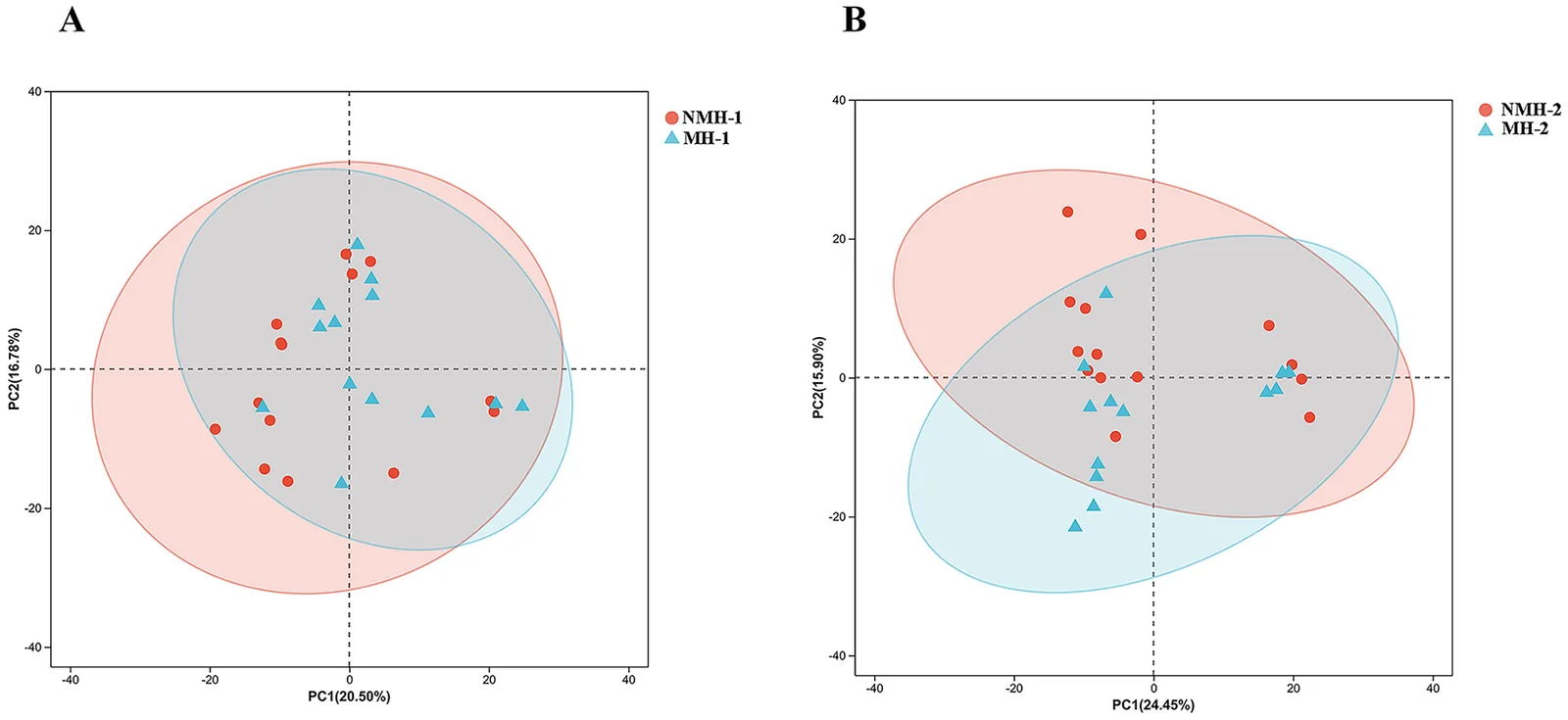
PCA analysis of transcriptome profiles between MH and NMH groups. **(A)** Baseline PCA; PERMANOVA: R^2^ = 0.037, *P* = 0.164. **(B)** Post-EEN PCA; PERMANOVA: R^2^ = 0.015, *P* = 0.912. PCA, Principal component analysis. NMH-1, non-mucosal healing group at baseline; MH-1, mucosal healing group at baseline; EEN, exclusive enteral nutrition; PC, principal component; NMH-2, non-mucosal healing group after EEN; MH-2, mucosal healing group after EEN.

### IL-17 signaling pathway dominates core enrichment after EEN intervention

For DEGs screening, DEGs were defined by the cut-off criteria of |log_2_FC| ≥ 1 and FDR ≤ 0.05. A total of 660 DEGs were identified between the MH-2 and NMH-2 groups, and 752 DEGs were identified between the MH-1 and MH-2 samples, which were visualized via volcano plots (Figures 3A, B). In total, 197 overlapping DEGs were screened out from the two comparison sets (Figure 3C). Subsequent KEGG pathway enrichment analysis of these overlapping DEGs confirmed that the IL-17 signaling pathway was the most significantly enriched pathway, involving nine core candidate DEGs (Figure 3D). Heatmap analysis further verified distinct expression patterns of these nine DEGs between MH-2 and NMH-2 specimens (Figure 3E). Collectively, these findings point toward the IL-17 signaling pathway as a candidate pathway showing correlation with EEN therapeutic responses.

**Figure 3:**
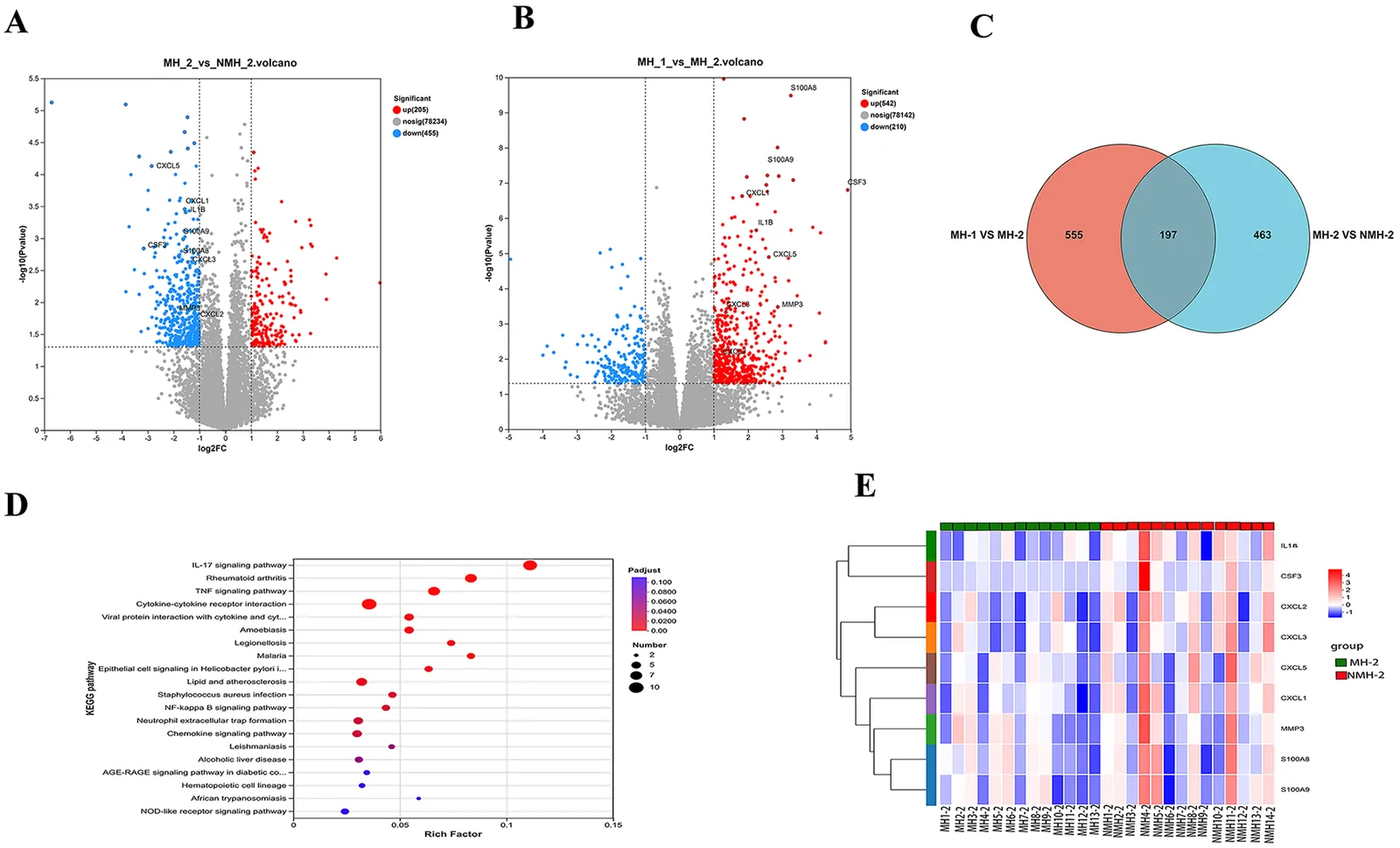
Bioinformatic analysis of the DEGs. **(A)** Volcano plot visually displaying DEGs between MH-2 and NMH-2 groups, a total of 660 DEGs (205 up-regulated and 455 down-regulated) were detected. **(B)** Volcano plot visually displaying DEGs between MH-1 and MH-2 groups, a total of 752 DEGs (542 up-regulated and 210 down-regulated) were detected. For volcano plots: red dots represent significantly up-regulated genes; blue dots represent significantly down-regulated genes; gray dots represent genes without significant expression changes. Vertical gray dashed lines indicate the log_2_ fold-change threshold (|log_2_FC| = 1); the horizontal gray dashed line represents the FDR significance threshold (FDR = 0.05). Gene labels mark nine core DEGs involved in the IL-17 signaling pathway. **(C)** Venn diagram showing the 197 overlapping DEGs identified in the two comparisons (MH-2 vs. NMH-2 and MH-1 vs. MH-2). **(D)** KEGG pathway enrichment analysis of these 197 overlapping DEGs. **(E)** Heatmap visualization of the 9 core DEGs associated with the IL-17 signaling pathway between the MH-2 and NMH-2 groups. DEGs, differentially expressed genes; MH-2, mucosal healing group after EEN; NMH-2, non-mucosal healing group after EEN; MH-1, mucosal healing group at baseline; KEGG, The Kyoto Encyclopedia of Genes and Genomes.

### IL-1β is identified as the core functional target DEG

qRT-PCR was further applied to validate the expression variations of the 9 candidate DEGs in intestinal mucosal tissues after EEN treatment. Only intestinal mucosal IL-1β expression was markedly downregulated in the MH-2 group relative to the NMH-2 group [0.78 (0.75) vs. 1.46 (2.41), *P* = 0.04, Figure 4A], while the remaining 8 IL-17 pathway-related DEGs exhibited no significant intergroup differences (Supplementary Table 2, which presents the median expression values and intergroup *P* values of CXCL1, CXCL2, CXCL3, CXCL5, CSF3, MMP3, S100A8 and S100A9 between MH-2 and NMH-2 groups after EEN intervention; all comparisons yielded *P* > 0.05 without statistical significance). A PPI network was constructed using the STRING database with an interaction confidence score > 0.4, and results revealed that IL-1β exhibited the highest degree centrality (degree = 30), identifying it as a predicted core hub gene from this bioinformatic network analysis. (Figure 4B). Spearman's rank correlation analysis revealed moderate positive correlations between intestinal mucosal IL-1β expression and three clinical indicators: SES-CD scores (*P* = 0.001, *r* = 0.42, 95% CI: 0.17 ~ 0.63), Lewis scores (*P* < 0.001, *r* = 0.65, 95% CI: 0.41~ 0.79) and wPCDAI values (*P* < 0.001, *r* = 0.51, 95% CI: 0.26 ~ 0.69) (Figures 4C–E).

**Figure 4:**
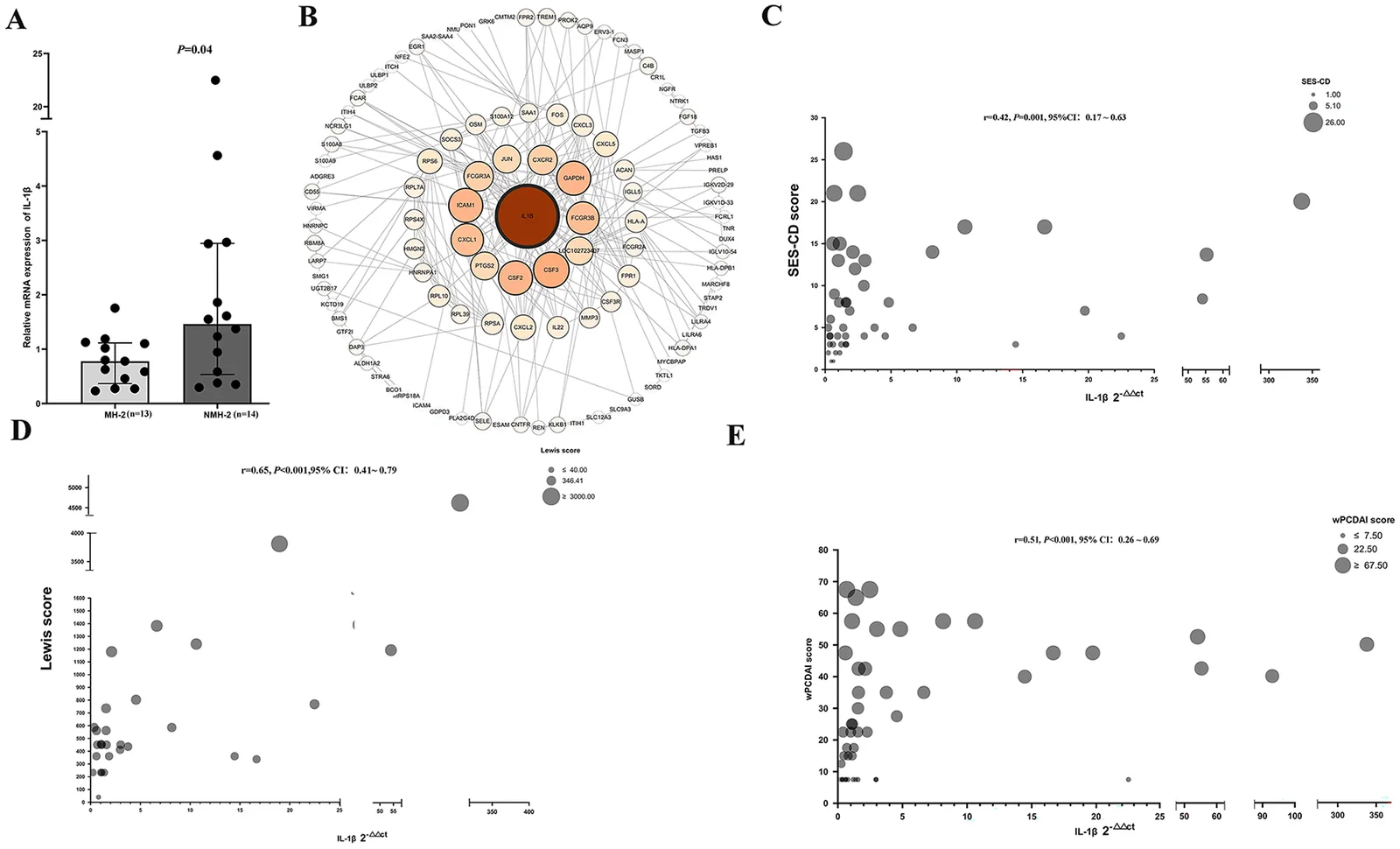
IL-1β was identified as the key target among DEGs. **(A)** The mRNA expression level of IL-1β in intestinal mucosal of the two groups at EEN completion was detected by qRT-PCR. **(B)** The processed PPI network of DEGs between MH-2 and NMH-2 groups after EEN. Larger node size, wider border and darker color indicate higher connectivity. **(C)** The correlation between intestinal mucosal IL-1β expression level and SES-CD score was analyzed using Spearman's rank correlation test. **(D)** The correlation between intestinal mucosal IL-1β expression level and Lewis score was analyzed using Spearman's rank correlation test. **(E)** The correlation between intestinal mucosal IL-1β expression level and wPCDAI was analyzed using Spearman's rank correlation test. DEGs, differentially expressed genes; EEN, exclusive enteral nutrition; PPI, protein-protein interaction; MH-2, mucosal healing group after EEN; NMH-2, non-mucosal healing group after EEN; SES-CD, simple endoscopic score for CD; wPCDAI, weighted Pediatric Crohn's Disease Activity Index.

### qRT-PCR quantification of IL-17 family expression post-EEN

qRT-PCR was used to detect intestinal mucosal expression levels of IL-17 family upon EEN completion. The results demonstrated that the expression of IL-17A [MH: 0.50 (1.42) vs. NMH: 2.58 (2.05), *P* = 0.03], IL-17RA (MH: 0.95 ± 0.37 vs. NMH: 1.34 ± 0.54, *P* = 0.04) and IL-17RB [MH: 1.18 (0.54) vs. NMH: 1.64 (1.32), *P* < 0.01) were significantly lower in the MH group than those in the NMH group.

## Discussion

In this study, we identified a close correlation between downregulated IL-1β expression, altered IL-17 signaling pathway activity, and EEN-associated mucosal healing in PCD patients. This prospective transcriptomic cohort study focusing exclusively on treatment-naive PCD patients receiving EEN as monotherapy to systematically characterize the transcriptional correlation between the IL-17,IL-1β molecular and endoscopic MH outcomes.

IL-17 is a key pathogenic cytokine and a well-recognized therapeutic target in various autoimmune and inflammatory disorders (11). Upon binding to its heterodimeric receptor, IL-17 recruits Act1, which interacts with TRAF6 to activate downstream signaling cascades, including the NF-κB and p38 mitogen-activated protein kinase pathways. These activated pathways subsequently promote the initiation and progression of inflammatory responses, including CD (15). However, IL-17 also plays a crucial role in maintaining intestinal barrier function by preserving tight junctions between epithelial cells and upregulating the expression of antibacterial proteins such as β-defensin and calcitonin (16, 17). Additionally, IL-17 can interact with fibroblast growth factor 2 in colonic epithelial cells to regulate the cell-growth-factor signaling pathway (18). This may explain why treatment with IL-17 inhibitors is associated with the onset and exacerbation of IBD (19, 20). Therefore, the clinical application of anti-IL-17 biological agents for disease treatment should be strictly regulated to ensure that residual IL-17 in the body can maintain the integrity of the skin and mucous membranes.

Over the past two decades, EEN has emerged as a highly effective treatment for the induction of remission in PCD. However, the molecular mechanisms through which EEN produces clinical benefit in CD remain incompletely defined. Our study identifies an observational association between reduced CD-associated inflammatory signatures and lower IL-17 signaling-related transcript levels after EEN, consistent with pre-clinical rodent data reporting that EEN could dampen Th17-type immune responses by reducing mechanical stress in colitis models (9). Notably, this observed transcript pattern differs from the complete IL-17-signaling suppression seen under IL-17-inhibitor treatment, a therapy known to worsen IBD in some cases. Our transcriptomic observations show coordinated reductions in IL-1β and IL-17-related transcript abundance following EEN, rather than total abolition of IL-17-related transcription. These transcript-level patterns raise the hypothesis that partial IL-17-pathway activity may be preserved to support intestinal mucosal integrity.

Published *in-vitro* and animal data indicate that IL-17 and IL-1β can act synergistically during inflammatory processes. According to existing mechanistic literature, IL-17 may drive IL-1β production via NF-κB activation, and IL-1β may in turn boost Th17 differentiation and IL-17 output, constituting a proposed inflammatory amplification loop (10, 21, 22). Prior multi-omics studies have nominated IL-1β as a hub molecule in CD and psoriasis (23, 24), which aligns with our bioinformatic prediction from mucosal transcriptomes. In our cohort, intestinal mucosal IL-1β transcript levels showed significant positive correlations with SES-CD, Lewis score and wPCDAI, supporting further exploration of IL-1β as a potential transcript-based biomarker for EEN-associated endoscopic mucosal-healing status in PCD.

Notably, the remaining eight IL-17-pathway-related DEGs did not reach statistical significance between groups. This observation could stem from our limited sample size, inter-patient heterogeneity among PCD subjects, or the possibility that these genes may exert secondary rather than central roles in transcriptomic signatures linked to EEN-associated mucosal healing, with IL-1β predicted as the central hub from network analysis. RNA-seq possesses high sensitivity for detecting subtle transcript shifts; hence modest expression alterations in these eight genes might be captured by RNA-seq yet fail to meet significance thresholds in qRT-PCR validation, given the more stringent quantitative readout of qRT-PCR assays.

We observed significantly lower mucosal transcript levels of IL-17A, IL-17RA and IL-17RB within the MH group relative to the NMH group. Previous data support a model whereby IL-17 may boost IL-1β production via NF-κB signaling, representing a well-described pro-inflammatory cascade implicated in CD-related intestinal inflammation (10, 22). In line with this existing framework, our data show that coordinated lower transcript levels of IL-17A, IL-17RA, IL-17RB alongside reduced IL-1β are associated with favorable EEN-related endoscopic outcomes. Integrating known biological functions of IL-17-family molecules (25), our data raise a hypothetical model (Figure 5): transcript-level shifts within the IL-17-signaling module after EEN may be linked to lowered IL-1β transcript abundance and reduced inflammatory transcript signatures.

**Figure 5:**
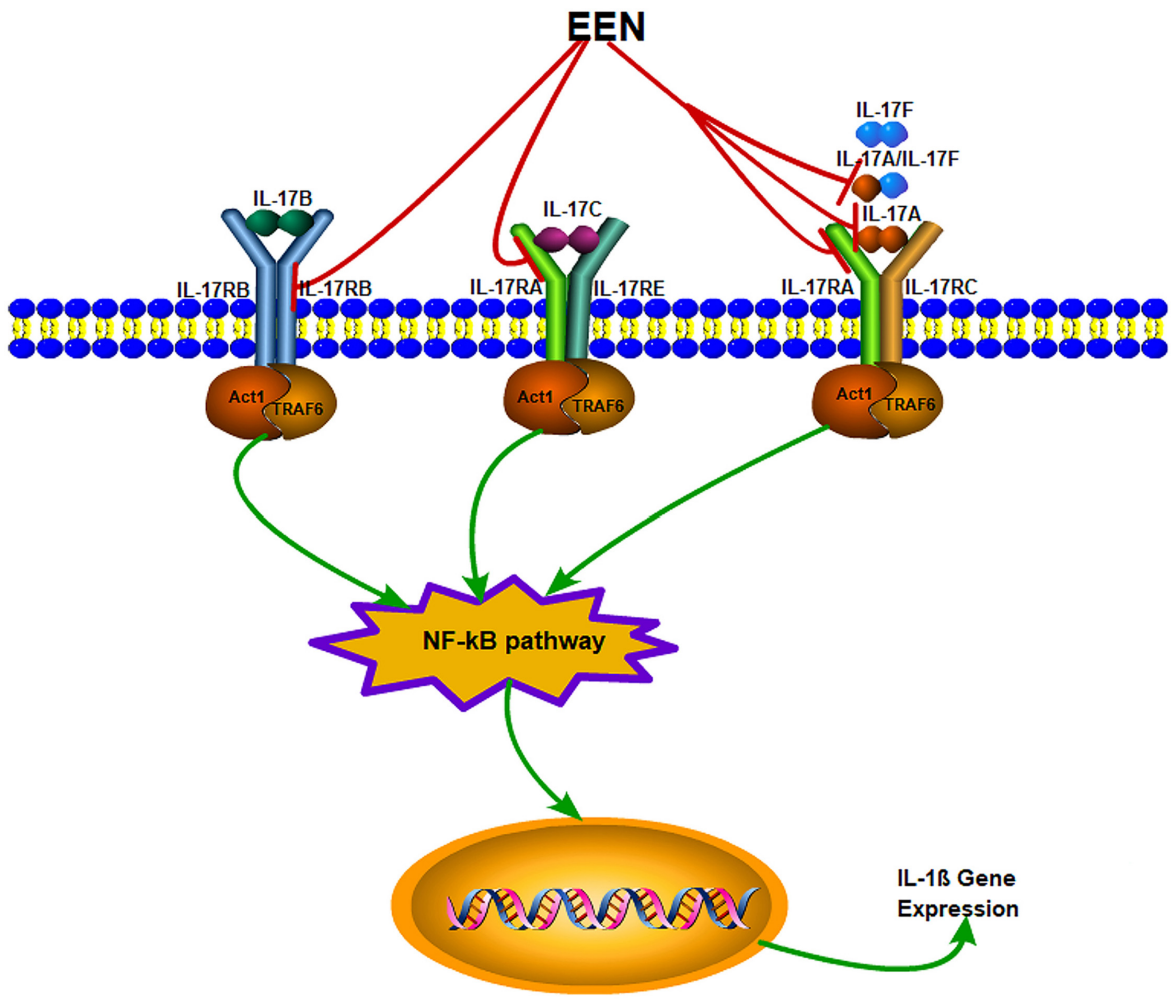
Potential association between altered IL-17 family levels and decreased intestinal mucosal IL-1β expression after EEN intervention (hypothetical model based on published literature). EEN, exclusive enteral nutrition.

We hypothesize that the transcriptomic shifts in IL-17-signaling components and reduced IL-1β transcript abundance observed among EEN-treated patients are probably not independent molecular events, but correlate with broader physiological changes accompanying EEN exposure, for example altered intestinal mechanical stress and shifts in gut-microbiota composition. Based on existing literature, EEN-related microbiota remodeling could lessen microbial mucosal stimulation and consequently dampen innate-immune signaling, which is a proposed upstream trigger for IL-1β expression. Future investigations are warranted to dissect whether microbiota shifts under EEN shape innate-immune outputs that correlate with IL-17-pathway and IL-1β transcript levels. Characterizing these correlative relationships may build theoretical context for understanding EEN effects in CD and inform optimization of nutritional regimens as well as candidate molecular markers for nutritional intervention. Clinically, measurement of mucosal IL-1β, IL-17A, IL-17RA and IL-17RB transcript levels merits further evaluation as potential markers for EEN-related therapeutic outcomes. Collectively, our work delineates key transcript-level molecular associations linked to EEN-related endoscopic responses in PCD and points toward candidate molecular signatures that could inform future personalized-EEN research directions.

This study has several limitations. First, it was conducted at a single center with a small sample of 27 participants, which may cause selection bias and insufficient statistical power to detect minor transcriptional differences; multi-center large-cohort studies are needed to validate our transcriptomic correlations. Second, we did not perform transcriptomic comparison between the NMH-1 and NMH-2 subgroups. Therefore, we cannot rule out the possibility that the nine identified genes represent a generic transcriptional response to EEN rather than a signature specifically associated with mucosal healing. Third, as an observational study based only on human mucosal transcriptome data, it cannot confirm causal links between EEN, suppressed IL-17 signaling and reduced IL-1β, nor clarify their complete molecular cascades. Fourth, only mRNA levels were measured via RNA-seq and qRT-PCR, without protein detection or cellular functional assays; further *in-vitro* and *in-vivo* experiments are required to verify their biological interactions. Fifth, mucosal tissues were collected only at baseline and post-EEN, lacking longitudinal transcriptomic data to track dynamic molecular changes during inflammation remission. Sixth, we did not analyze the mediating effect of gut microbiota, and integrated transcriptome-microbiome multi-omics work is warranted to identify microbial upstream regulators of mucosal immune signals.

## Conclusion

In conclusion, our observational study identifies associations between markers of dampened IL-17 signaling activity, diminished IL-1β mucosal secretion, and favorable clinical outcomes following EEN among children with PCD. These correlative findings cannot establish causal relationships and require further independent validation in larger cohorts and functional experimental studies, yet they provide novel clues for future investigations into potential molecular pathways linked to EEN intervention.

## Data Availability

The datasets presented in this study can be found in online repositories. The names of the repository/repositories and accession number(s) can be found below: https://ngdc.cncb.ac.cn/bioproject/browse/PRJCA065778.
