# Supplementary material for: Therapeutic response to exclusive enteral nutrition correlates with suppressed IL-17 signaling activity and diminished IL-1β secretion in pediatric Crohn's disease
Source: Front Nutr. 2026 Sep 14;13:1901066. doi: 10.3389/fnut.2026.1901066 (PMC13618058; doi:10.3389/fnut.2026.1901066)
Supplement: Supplementary file 1 [file Table_1.docx]

| **Primer** | **Primer sequences (5'**→**3')** | |
| --- | --- | --- |
| IL-1β | F: GCGGCATCCAGCTACGAAT  R: AAGCCTCGTTATCCCATGTGTC | |
| CXCL1 | F:AGCTTGCCTCAATCCTGCAT  R: GCTCCTCCAGCCTCTAGAGT | |
| CXCL2 | F:CAAACCGAAGTCATAGCCACA  R: TCCTTCAGGAACAGCCACCA | |
| CXCL3 | F: CGCCCAAACCGAAGTCAT  R: TTGGTGCTCCCCTTGTTCAGTA | |
| CXCL5 | F:CTCCACCCAGTTCAGGAACC  R:CGTTCTTCAGGGAGGCTCTG | |
| CSF3 | F: TGCTGCTCGGACACTCTCTG  R: GAGCCCCTGGTAGAGGAAAAG | |
| MMP3 | F:GTAGCAGCACTGGGGCTTAA  R: TTTCCTCCACTTCGGGATGC | |
| S100A8 | F: AGGAGCTGAGCCAACAATCC  R: AACAAGGTCTCCAGCTCAGC | |
| S100A9 | F: CGCAGTTTGGGTTGACAAGG  R: GGTGCTCTCCATGTGCTCTT | |
| IL-17A | F: GTATTTCTGGACCGTGGGCA  R: AGTGCTGTTGGTGCTACTGG | |
| IL-17B | F: ATCTTTGGGAATGGCAGGGG  R: GGCCCGGTTTTCCTTGAAAC | |
| IL-17C | F: GGAGATACCGGTGAGGACCT  R: CGTGCATCGATACAGCCTCT | |
| IL-17D | F:CCGTGATGTGGACACAGTGA  R:CCCTCAGCACTGCCTTTGTA | |
| IL-17F | F: TTCTCTGTGCCTCCGTTTCC  R: TCCTATCTGCCTGCACCTCT | |
| IL-17RA | F: CCCACCCCTTTACCCATCAC  R: CCACCACCAAGCAAGCAATC | |
| IL-17 RB | F: CCAAGGTTGGGATAGCTGCA  R: AGCACCAAAAACACCCTGGA |  |
| IL-17 RC | F: CCTTAGCTTTGGCCAGGTGA  R: GGACTGACTCTGGGGAGGAT |  |
| IL-17 RD | F: CTCCGGGAAAAGAAACGGGA  R: GCAAGGTTTCCCAGGCTTTG |  |
| IL-17 RE | F: GACTGTGCACCTAACCCCTC  R: CACAAACAGCGGGAACAGTG |  |
| GAPDH | F: GGAAGCTTGTCATCAATGGAAATC  R: TGATGACCCTTTTGGCTCCC |  |

Supplementary table 1 Primer sequences for quantitative reverse transcription polymerase chain reaction in this study

| DEGs | MH-2 | NMH-2 | *P* |
| --- | --- | --- | --- |
| CXCL1 | 0.34(0.42) | 0.67(1.33) | 0.20 |
| CXCL2 | 0.36(0.34) | 0.55(1.08) | 0.57 |
| CXCL3 | 0.13(0.26) | 0.34(0.59) | 0.23 |
| CXCL5 | 2.08(2.28) | 2.03(27.28) | 0.43 |
| CSF3 | 0.75(1.09) | 1.08(0.45) | 0.71 |
| MMP3 | 0.9(6.41) | 2.58(5.70) | 0.43 |
| S100A8 | 6.83(8.70) | 8.36(55.85) | 0.33 |
| S100A9 | 10.95(18.75) | 25.88(108.04) | 0.15 |

Supplementary table 2 compares the mRNA expression levels of the other 8 IL‑17 pathway‑related DEGs in the intestinal mucosa following EEN intervention.

Abbreviations: DEGs, differentially expressed genes; EEN, exclusive enteral nutrition; MH-2, mucosal healing group after EEN; NMH-2, non-mucosal healing group after EEN.
